# Hydrogenases and the Role of Molecular Hydrogen in Plants

**DOI:** 10.3390/plants9091136

**Published:** 2020-09-02

**Authors:** Grace Russell, Faisal Zulfiqar, John T. Hancock

**Affiliations:** 1 Department of Applied Sciences, University of the West of England, Bristol BS 16 1QY, UK; Grace2.Russell@live.uwe.ac.uk; 2 Institute of Horticultural Sciences, Faculty of Agriculture, University of Agriculture Faisalabad, Faisalabad 38040, Pakistan; ch.faisal.zulfiqar@gmail.com

**Keywords:** antioxidants, hydrogen rich water, nitric oxide, reactive oxygen species, stress responses

## Abstract

Molecular hydrogen (H_2_) has been suggested to be a beneficial treatment for a range of species, from humans to plants. Hydrogenases catalyze the reversible oxidation of H_2_, and are found in many organisms, including plants. One of the cellular effects of H_2_ is the selective removal of reactive oxygen species (ROS) and reactive nitrogen species (RNS), specifically hydroxyl radicals and peroxynitrite. Therefore, the function of hydrogenases and the action of H_2_ needs to be reviewed in the context of the signalling roles of a range of redox active compounds. Enzymes can be controlled by the covalent modification of thiol groups, and although motifs targeted by nitric oxide (NO) can be predicted in hydrogenases sequences it is likely that the metal prosthetic groups are the target of inhibition. Here, a selection of hydrogenases, and the possibility of their control by molecules involved in redox signalling are investigated using a bioinformatics approach. Methods of treating plants with H_2_ along with the role of H_2_ in plants is also briefly reviewed. It is clear that studies report significant effects of H_2_ on plants, improving growth and stress responses, and therefore future work needs to focus on the molecular mechanisms involved.

## 1. Introduction

There is an increasing weight of evidence showing molecular hydrogen (H_2_) has effects in biological systems, in both animal [1] and plant species [2]. When considering an ever-increasing global population, and the growing obligation we have to feed each individual, the requirement for inexpensive and sustainable treatments which enhance the quality and longevity of produce will be necessary to prevent shortages of essential farmed foods. In this regard, treatment with H_2_ has been described as being beneficial for a range of human diseases [3,4], whilst in agriculture, application of H_2_ has been demonstrated to increase both crop health and yield [5], important factors that may prove beneficial for the arable and cattle-feed industries in particular. To illustrate, evidence shows that H_2_ is able to mediate root development and stress responses in plants, in response to heavy metals [6] and drought [7] in particular. It can also be used for improving post-harvest storage of crops, for example with kiwifruits [8]. Therefore, how organisms such as plants can be exposed to H_2_, and how they respond to it, is important to understand and may lead to better treatments and better yields in the future.

In light of ongoing research it is becoming clear that H_2_ should be seen as part of a suite of small reactive molecules that can influence and control cellular function. It has long been known that reactive oxygen species (ROS), such as superoxide anion (O_2_^−^), hydrogen peroxide (H_2_O_2_) and hydroxyl radicals (OH), are produced in cells and can impact activities in the intracellular and extracellular environs, during stress responses as an example [9]. Of further significance are the reactive nitrogen species (RNS), such as nitric oxide (NO) and peroxynitrite (ONOO^−^) [10]. Hydrogen sulfide (H_2_S) too, is an important signalling molecule [11], often produced, along with ROS and RNS, during stress responses [12].

As these molecules can be generated by plant cells concomitantly, both temporally and spatially, it is likely that there would be an interplay between them [13], and also, interactions with H_2_. This crosstalk could, of course, be bidirectional wherein H_2_ may interfere with NO signalling, for example. Alternatively ROS and NO may also modify H_2_ metabolism. In either case, signalling events would be affected, and this would influence both short-term and long-term cellular activities.

H_2_ is an extremely small (MW 2.016 g/mol) and relatively inert molecule. As a consequence, it is hard to envisage how it can be perceived by a classical receptor protein, or how it could partake in the control of proteins through covalent modifications, as has been found for NO effects, typically through *S*-nitrosation [14]. However, H_2_ has been shown to have effects through the selective removal of reactive oxygen species (ROS), in particular hydroxyl radicals [15], and the scavenging of RNS, in particular peroxynitrite. H_2_ is also known to have effects through action on haem oxygenase enzymes (e.g., HO-1) [16]. It has also been mooted that the physical properties of H_2_ may mediate some of the effects seen in higher plants and animals [17]. However, we are currently far from a full understanding of how H_2_ interacts with cellular components and influences cellular activity, and a great deal of research into the molecular mechanisms of the interplay between molecular hydrogen and cellular systems will be required if we are to elucidate such complexities.

Cells may be exposed to H_2_ from both endogenous and exogenous sources. Exogenous sources, such as the arrival of H_2_ from the environment, are important to consider, especially as this may be the way H_2_ is used as a medication or agricultural treatment. However, many organisms are known to contain discrete hydrogenase enzymes responsible for the reversible oxidation of molecular hydrogen. Such enzymes are typically classified on the basis of their metal chelation properties e.g., Fe, FeFe, and NiFe [18], which may either generate or remove molecular hydrogen in cellular systems. In animals, the gut microflora is also an important source of H_2_ [19]; however, non-gut bacteria may also contribute to accumulation of H_2_ in biological systems [20]. Here, bacteria as a H_2_ source for plants is briefly discussed.

In this review, the manner in which plants can be exposed to molecular hydrogen are explored, along with a discussion of how the enzymes involved may be impacted on by reactive signalling entities such as nitric oxide. The effects that have been reported in plants are also discussed, although the molecular mechanisms underpinning the action of H_2_ is not clear. Future work in this area may open the way for beneficial treatments of plants [5], a topic which will be briefly discussed here as well.

## 2. Hydrogen Production by *Chlamydomonas reinhardtii*

Algae are convenient and well-used models for exploring the biochemistry and physiology of higher plants. In this regard, the species *Chlamydomonas reinhardtii* is commonly used [21]. *C. reinhardtii* is a single-celled alga that has two flagella and a large single chloroplast occupying the majority of intracellular space. Although not a higher plant, it is pertinent here as it has the capacity to produce molecular hydrogen [22]. *C. reinhardtii* contains two [FeFe]-hydrogenases (HYDA1 and HYDA2; 497 and 505 amino acid lengths, respectively) [23]. Interestingly, all algal species studied that possess hydrogenase activity contain two such enzymes, which are likely to have formed from gene duplication events. Here, the hydrogenases are stromal located enzymes which are coupled to the electron transport of photosynthesis, accepting electrons from ferredoxin [24]. In the short term, electrons can be supplied via Photosystem II (PSII), but generated oxygen will soon inhibit the hydrogenase activity. Over the longer term, such activity can be driven from a NAD(P)H-plastoquinone-oxidoreductase [25]. Factors here that are noteworthy include the aforementioned hydrogenase genes, which are only expressed in the absence of oxygen [26], and that H_2_ production is increased in the absence of CO_2_. Hydrogen production is also light dependent but H_2_ is increased in low sulfur [25]. When considering these observations, it appears that production of H_2_ is dependent on the action of PSII, and the subsequent generation of starch, which is used as an electron donor [27]. However, mutants that lack Rubisco also do not accumulate starch, but these cells can produce H_2_, probably through the action of PSII [22]. When working with algae, Torzillo et al. [28], recognized that sulfur-depleted *C. reinhardtii* cells produced H_2_ in vivo at a rate in the order of 10 nmol.hr^−1^.µg Chlorophyll^−1^. Production in a D1 (a protein in the PSII center) mutant resulted in considerably more H_2_, mainly because generation was prolonged for longer [28]. In addition to this, a study designed by Philipps et al. [29] investigated what would happen under nitrogen deficiency, and although starch was accumulated when sulfur was deficient—which should increase H_2_—it was found that H_2_ generation was low [29]. It was suggested that this was because of increased cytochrome *b_6_f* complex degradation and reduced amounts of ferredoxin.

It is clear, therefore, that some lower photosynthetic organisms such as *C. reinhardtii* can produce significant amounts of H_2_ under certain environmental conditions. The probable function is to aid in the coordination of electron flow during times of altered environmental conditions, such as the rising presence of oxygen. Sustained H_2_ generation is probably wasteful to the cells [23]. However, such H_2_ production, if it can be increased and sustained will be of interest as a method for generating H_2_ gas as a biofuel [30,31].

A BLAST search using the HYDA1 amino acid sequence shows that many other algae also have homologous hydrogenase proteins, including *Tetrabaena* *socialis, Gonium pectoral, Volvox carteri* and *Chlorella* (data not shown). Clearly, H2 metabolism in green algae is a common occurrence, not exclusive to *C. reinhardtii.*

### Possible Control of Algal Hydrogenases

As discussed in more depth below, H_2_ may have effects on ROS and NO metabolism. Therefore, it may be that a feedback mechanism would allow effects of ROS and NO in the control of hydrogenase activity. It was reported a long time ago that hydrogenases could be inhibited by NO [32]. NO is likely to act on proteins by covalently modifying cysteine residues through *S*-nitrosation to create the -SNO group [33], or through modification of tyrosine residues forming a nitrotyrosine group [34]. Evidently, HYDA1 has eleven cysteine residues, whilst HYDA2 has nine, with six common to both (data not shown), so there is a possibility of thiols being able to be covalently modified by ROS or NO, assuming such thiols are accessible and not buried in the protein structure. Using the iSNO-PseAAC prediction tool [35] to find possible positions of -SNO groups in HYDA1, Cys54, Cys170, Cys191, and Cys225 are suggested to be *S*-nitrosated. Using the HYDA2 sequence Cys17, Cys33, Cys194, and Cys228 are suggested to be *S*-nitrosated. The common ones shared by these two sequences are Cys191/194 and Cys225/228 (data not shown), perhaps suggesting that they may be significant for NO modification. However, it has been suggested that the three-dimensional structure around the nitrosated cysteine is important, rather than the amino acid sequence directly next the cysteine [36]. It has also been noted that for the formation of the -SNO group the presence of a (IL)-X-C-X-X-(DE) motif is favourable [37]. Both the HYDA1 and HYDA2 sequences are devoid of this sequence (data not shown), and perhaps therefore are not able to be *S*-nitrosated.

Of course, it may not be the amino acid sequence which is the target for control of hydrogenases. It has long been known that NO inhibits such enzymes [32], but more recently the NiFe hydrogenase of *Desulfovibrio fructosovorans* has been studied. Here, Ceccaldi et al. [38] demonstrated that the metallic elements were the target of NO, and that NO inhibited the NiFe active site whilst also irreversibly damaging the iron–sulfur centers [38]. Therefore, for NO action there is no specific requirement for a thiol modification at all.

No amino acid motif has been reported for ROS modification of thiols, so this would still be a matter for debate. Similarly, despite an extensive study on *S*-persulfidation in *Arabidopsis thaliana* there was no consensus of a target amino acid sequence for H_2_S mooted [39]. It has been suggested that sites that are *S*-persulfidated are similar in nature to those *S*-nitrosated [40], with the three-dimensional aspects of the thiol of vital importance, and with glutathione persulfide (GSSH) mediating the amino acid modification.

In a similar way, it might be possible to predict if such proteins are tyrosine nitrated. A nitration motif has been suggested [41] with the sequence:

-DKDADGWISPA**Y**AK-

where the target tyrosine is underlined and bold. However, even though the HYDA1 sequence has nine tyrosine residues, there is no similarity in their surrounding sequences to the nitration sequence given here. HYDA2 differs in that it has eight tyrosine amino acids, and again there is no evidence that tyrosine nitration should be predicted (data not shown). Interestingly, "Prosite" [42] shows no prediction of tyrosine phosphorylation either (data not shown).

Others have designed a motif which may be glutathionylated [43]. This sequence is similar to the nitration sequence, both being based on EF-hand motifs. Here the sequence is:

-DKDADGW**C**G-

where the target cysteine is underlined and bold. Again, no similarly to this sequence is found in either the HYDA1 and HYDA2 amino acid sequences, which is not surprising as Prosite [42] does not predict the presence of a EF-hand consensus sequence either. This latter point might cast doubt on any control through Ca^2+^ metabolism too, which is important in many plant cell responses [44], although there are other methods of Ca^2+^ ion control other than the action of EF-hands.

## 3. Hydrogenases of Higher Plants

It has been known for a long time that hydrogen is metabolized by higher plants and that (FeFe)-hydrogenases exist in eukaryotic species, including plants [45]. These metallo-protein complexes appear to be able to catalyze both forward and backwards reactions, effectively removing or generating H_2_. They also seem to be involved in the appropriate biosynthesis of (Fe-S) clusters and the sensitivity to O_2_. Knockouts of encoding genes leads to poor plant development, with these enzymes appearing to be involved in the control of the cell cycle and sugar metabolism [46], as well as transcription control and in stress responses [47]. Eukaryotic hydrogenases are often referred to as NAR (nuclear architecture related) or GOLLUM (different oxygen levels influences morphogenesis) proteins. For example, in plants there is GOLLUM1 in *Medicago truncatula* [48] and AtNAR1 in *Arabidopsis* [49], although currently such naming appears to be interchangeable.

It is tempting, as with the hydrogenases in *C. reinhardtii*, to suggest that redox signalling molecules may have an impact on the action of higher plant hydrogenases. As with the HYDA1 and HYDA2 sequences above, if such enzymes are controlled by the presence of ROS, NO, or H_2_S, then thiol side groups may be the targets for modification. This may, for example, be for covalent modification with ROS or NO, but neither polypeptide sequence contains any -SNO motifs ((IL)-X-C-X-X-(DE) [37]) suggesting that control by NO by this means is unlikely. A brief analysis of the Arabidopsis AtNar1 sequence (Accession number: NM_117739) shows 13 cysteine residues. When aligned with the ferredoxin hydrogenase from *Artemisia annua* (sweet wormwood or qinghao: Accession number: PWA71961) there is 69% identity in the sequence. Interestingly, all but one of the cysteine residues is conserved, thereby evolution would suggest that they are retained for a reason.

**Figure 1:**
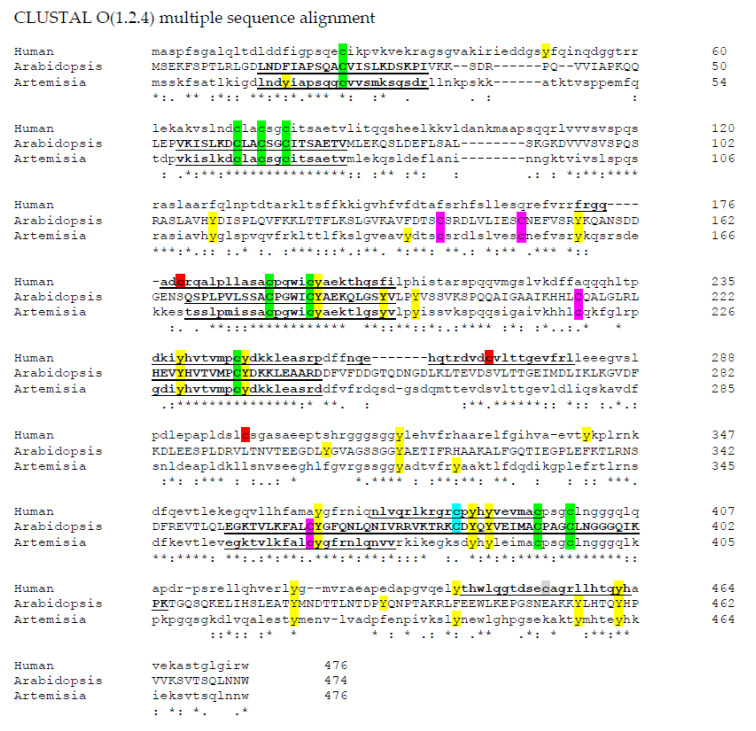
Amino acid sequence alignments of hydrogenases. *Clustal Omega* [50] was used to align three hydrogenase sequences: human cytosolic iron-sulfur assembly component 3 isoform 1(Accession number: NP_071938.1); AtNar1 (Accession number: NP_567496.4); ferredoxin hydrogenase from *Artemisia annua* (Accession number: PWA71961). C: Common to all sequences; C: common to just plant species; C: Common to human and Arabidopsis; C: unique to human; and Y: tyrosine residues. Sequences predicted to become -SNO using the iSNO-PseAAC prediction tool [35] are underlined and in bold. -SNO: nitrosated thiol. * Means total consensus, : means conserved changes, . means less conserved and a gap is not conserved.

Aligning these plant sequences with the sequence for the human cytosolic iron-sulfur assembly component 3 isoform 1 (Accession number: NP_071938.1), using *Clustal Omega* [50], there are still nine conserved cysteine residues. Surprisingly, Cys 380 is conserved in Arabidopsis AtNAR1 but not in the Artemisia sequence, yet it is contained in the human sequence (Figure 1). The human sequence also contains three cysteine residues not found in the plant sequences, but still they do not fall in a -SNO conserved region. However, putting the sequence through the iSNO-PseAAC prediction tool [35] gives the data shown in Table 1. Four conserved cysteine residues in the three sequences investigated were predicted to be modified by NO. Using the Arabidopsis sequence numbers, these were Cys24, Cys177, Cys233, and Cys362. Interestingly, two of these are conversed also in the human sequence: Cys177 and Cys233 (Table 1 and Figure 1). Such conservation across a wide range of species suggests that they have a possible significant role and it may be that they are there for control through NO signalling. This is, of course, with the caveat that it has been suggested that the three-dimensional orientation of the amino acids is more important than the sequence in the region [36]. Furthermore, as discussed above, it is likely that the metal prosthetic groups are the target for NO, rather than the amino acid thiol side chains [38].

The three-dimensional orientation of amino acids may be important for S-persulfidation of these proteins as well [40], although there appears to be no evidence in the literature of hydrogenases being covalently modified in this way. This being said, it is known that hydrogenases are inhibited by H_2_S [51], perhaps by attack on the metal center, as suggested for NO [38].

All three sequences (Human, *Arabidopsis*, and *Artemisia*: Figure 1) have eight conserved tyrosine residues. It is possible that they may be used for nitro-tyrosine formation, but there is no evidence of the conserved region used by Urmey and Zondlo [41]. In a similar way, looking for the cysteine residues which may be glutathionylated [43], there is no evidence of this sequence being in the hydrogenase amino acid sequences investigated here (Figure 1). From what can be considered a rather naïve point of view, using bioinformatics, it is therefore possible that hydrogenases in higher organisms are inhibited by NO through thiol modification, and possibly by ROS, but as yet there is little evidence that such control is significant.

## 4. Influence of Bacteria on Hydrogen Availability to Plants

As well as from endogenous sources, the bioavailability of H_2_ can be influenced by external factors. Naturally, this may be from other organisms [52], such as bacteria [53] or fungi [54], for example. It has also been suggested that bacteria in the soil which oxidize H_2_ can promote the growth of plants [55]. Nutrient availability, and factors which affect it, are crucial for plant growth [56], as well affecting mineral accumulation in crops [57]. Clearly plant/soil interactions are crucial for healthy plants [58].

As far back as 1937, Wilson and Umbreit [59] reported the effects of H_2_ on nitrogen fixation. Root nodules containing a symbiont are known to evolve H_2_; however, this appears to be detrimental to nitrogen fixation. Endogenously, H_2_ is a by-product of nitrogenase activity [60], with approximately 50% of the electrons being used to fix nitrogen, the rest being used to produce H_2_. This is then lost to the environment [61], possibly accounting for some of the benefits of crop rotation [62].

H_2_ oxidizing bacteria are known to be associated with many plants and such organisms as streptomyces in particular contain an (NiFe)-hydrogenase. To illustrate, Kanno et al. [63] inoculated seedlings of *Oryza sativa* and *Arabidopsis thaliana* with streptomyces and found that the bacteria were taken into the plant tissues and that their H_2_ oxidizing activity continued. Likewise, treatment of soils with H_2_ can alter the populations of microorganisms which may be found there [2], and this can also have an influence on plant growth.

It has long been known that some hydrogenases can be inhibited by NO [32], and that hydrogenase enzymes can also be inhibited by O_2_, CO, and acetylene [64]. The inhibition by NO was originally reported for *Proteus vulgaris* in 1954 [32]. Since then, it has been determined that NO may influence the [Fe-S] clusters [65]; however, it should be noted that the interaction of (NiFe)-hydrogenase with NO is much more complex than this [38]. Nevertheless, these studies indicate that NO can indeed influence hydrogenase activity. Furthermore, such hydrogenases have also been identified as being inhibited by H_2_S [51]. As plants are known to produce both NO [66] and H_2_S [67], there is potential for an interaction between gaseous signalling species, this would effectively modulate H_2_ metabolism and any subsequent downstream effects.

## 5. How to Treat Plants with Molecular Hydrogen

Molecular hydrogen is a gas, and so the obvious way to treat an organism is to use it as an aerosol. This approach is used to treat animals which can breathe the gas in, and is often utilized in the medical arena [3]. However, for plants, unless this is carried out in an enclosed space this would not be a practical approach. In addition, H_2_ in its gaseous form is also highly flammable, and so safety issues need to be a primary consideration. Therefore, for large scale treatments, including agriculture, an alternative approach needs to be taken.

Often hydrogen is given to an organism as a saturated solution. This may be what is referred to as hydrogen–rich water (HRW), as used by Liu et al. [68]. To produce this water can be bubbled with hydrogen gas. The solubility of H_2_ is relatively low, and it has been suggested, using Henry’s Law, that a saturated solution has a concentration of H_2_ of approximately 1.6 mg·L^−1^ (equivalent to 0.8 mM) [69]. However, H_2_ will rapidly enter the vapor phase (atmosphere) so a saturated solution would not retain this concentration of H_2_ for long. An alternate method for producing HRW is to use a magnesium-based tablet. With many commercial products available, it is relatively easy to produce a water-based solution enriched with H_2_. To treat plants, such a solution can be watered onto the soil or sprayed over the foliage. However, if H_2_-generating tablets are used, it should be considered that there will be by-products left over in the solution, including additives included by the tablet manufacturers. In addition, of possible important use, commercially available products that utilize methylene blue-based techniques and quantify the dissolved hydrogen in solution, have been developed [70]. Therefore, an estimation of dissolved H_2_ can be obtained before any solution is used, which will help in ensuring preparations are suitable before treatments are given.

Alternatively, a saline, or salt solution can be enriched, in what is referred to as hydrogen-rich saline (HRS). Although useful for animal research [71], this is less likely to be useful for plant treatments, unless high salt is needed or being studied, such as carried out by da-Silva et al. whilst looking at the effects of H_2_S [72].

From a biological viewpoint the treatment of plants with H_2_ should be relatively safe. There are few, if any, detrimental effects of H_2_ on biological systems. However, from a pragmatic point of view, it has to be remembered that H_2_ is highly explosive, especially the presence of atmospheric oxygen. Therefore large-scale spraying of crops may require some safety measures.

For an overview on the application of molecular hydrogen, along with the use of other reactive molecules used in signalling, a review by Hancock [73] is recommended.

## 6. H_2_ Effects on Plants

H_2_ is involved in a range of physiological responses in plants, which is one of the reasons why it has been proposed to be a useful tool for agriculture [20]. As only some highlights are discussed here, an in-depth review by Li et al. [2], which focusses on the multiple roles of H_2_ within plant systems is recommended.

An early study looked at the uptake of H_2_ by leaves [74]; however, as previously discussed, plants produce molecular hydrogen by endogenous means as well. To exemplify, in experimental studies using tomato plant seedlings, endogenous H_2_ generation was stimulated by treatment with naphthalene-1-acetic acid (NAA; an auxin analogue), whilst H_2_ generation was later reduced by the inhibition of auxin transport with *N*-1-naphthyphthalamic acid (NPA). These experiments have demonstrated that an increase of biologically available H_2_ can promote lateral root development with both NAA and H_2_ promoting the generation of NO, hypothesized to mediate the effects seen [75]. Additionally, Lin et al. [16] have also reported that the application of HRW could regulate root development in cucumber plants. Here, the response was thought to be mediated through the enzyme HO-1. Post-harvest, treatment with hydrogen may be beneficial too. It was found that treatment of kiwi fruits with HRW delayed ripening. Lipid peroxidation was lowered and activity of superoxide dismutase was increased [6].

In a similar manner to that seen with ROS, NO and H_2_S, H_2_ has been implicated in mediating stress responses in plants. The inhibition of root elongation by aluminum was alleviated by H_2_ in alfalfa [76]. Interestingly this response also involved NO signalling. Others too have reported H_2_ effects on aluminum stress [77]. Here, plant hormones such as gibberellic acid (GA) and abscisic acid (ABA) were implicated, along with mechanisms which involved miRNA and gene expression. Zeng et al. [78] showed that ABA, jasmonic acid (JA), and ethylene all increased H_2_ generation in rice. They also showed that H_2_ had significant effects on the levels of antioxidants, and on gene expression. Such mechanisms were shown to bestow stress tolerance during salt and drought stress [7]. This is also evidenced in additional research that supports the supposition that H_2_ treatments can alleviate salt stress [79,80]. H_2_ treatment has also been shown to mitigate other stresses as well, for example, HRW reduced cadmium ion toxicity in *Medicago sativa* [6] and mercury ion stress in alfalfa [81].

Earlier in this paper the interplay between H_2_ and other reactive signalling systems has been described, how excess ROS can cause oxidative stress, and which molecules may be induced by the presence of other stress initiators, such as heavy metals [82]. Of pertinence here is an empirical study conducted by Jin et al. [83], utilizing alfalfa seedlings as a model. Here, H_2_ gas was shown to increase the tolerance to oxidative stress induced by the presence on paraquat (which increases the accumulation of ROS) [83]. This effect was mediated by the haem oxygenase system in these plants. Hydrogen treatment has also been shown to alleviate oxidative stress in other organisms too, such as animalia and human beings [84], although the mechanism of action remained elusive. Clearly, H_2_ has effects on some ROS and RNS species, such as hydroxyl radicals [15], but this is unlikely to be the only mode of action of H_2_ as upregulation of antioxidant genes is another, as yet not well understood, example of the beneficial effects attributed to H_2_. Some of the effects of H_2_ on plants discussed here are summarized in Table 2.

## 7. Conclusions and Perspectives

There is a growing body of evidence that molecular hydrogen is perceived by organisms and has beneficial effects. Lower plants such as *C. reinhardtii* are known to produce substantial quantities of H_2_, so much so that their use as a source of H_2_ to be used as a biofuel has been suggested [30,31]. Higher plants also contain hydrogenases, which catalyze the reversible oxidation of H_2_. H_2_ is likely to be present in cells spatially and temporally with other reactive molecules used in cell signalling, such as ROS, NO, and H_2_S. Therefore, the interplay between H_2_ enzymes and metabolism will need to take this into account in future enquiries. Spatial and temporal measurements of all the reactive molecules involved in signalling need to be undertaken, probably requiring new fluorescent probes. Only by knowing where and when all relevant players in the regulatory orchestra are accumulated, will a full understanding of the manner in which they give a coordinated response be gained.

Hydrogenase enzymes are known to be inhibited by NO [32]. Even though -SNO formation sites can be predicted in hydrogenase sequences using algorithms such as iSNO-PseAAC [32], there is little, if any, experimental evidence that they are used in such a way, rather that the metal centres are likely targets [38]. Similarly, hydrogenases are known to be inhibited by H_2_S [52], but again there is little evidence of such proteins being *S*-persulfidated. Future experimental work should be focused on clarifying if there are any effects of NO, ROS, and H_2_S on the accumulation of H_2_ in plant cells.

**Figure 2:**
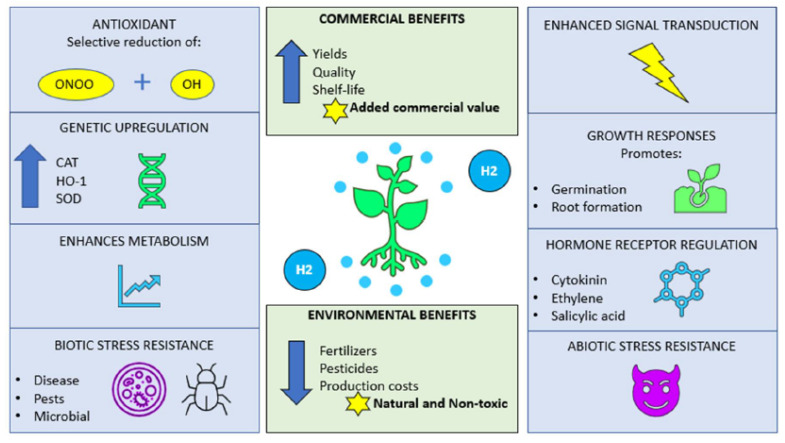
Possible mechanisms by which H_2_ acts in as a protective agent within plant systems. Several ways are shown by which H_2_ may act, whilst highlighting the benefits for both agricultural and environmental sustainability. ONOO: peroxynitrite; OH: hydroxyl radical; CAT: catalase; HO-1; haem oxygenase-1; and SOD: superoxide dismutase.

The bioavailability of H_2_ for a plant will also be influenced by the environment, perhaps by associated organisms. However, it is now well known that climate change-induced abiotic stress events such as droughts, floods, or soil infertility in particular are exerting a negative influence on agricultural yields. With the frequency of such challenging situations increasing, there is an inescapable need to search for yield enhancing strategies to meet the target of food security for feeding the growing human population. Another aspect that favours the utilization of non-toxic substances such as molecular hydrogen, is the current and continued usage of chemical fertilizers for enhancing yields. These practices are not sustainable when considering long-term future plans as the chemicals used in these products are recognized as being environmentally destructive, on land and in aqueous regions, where there is a threat to life caused by noxious chemicals leaching from surrounding farm lands. Figure 2 gives a brief synopsis of how molecular hydrogen can effectively increase the quality, yields, and longevity of produce whilst reducing both production and environmental costs.

Of particular interest to plant science is how the application of H_2_ can be carried out in a commercial setting [20], and how this may be made sustainable and safe, considering hydrogen is highly inflammable. Spray treatments with HRW are probably the most convenient, cost-effective, and practical methods, where delivery can theoretically be applied either onto the soil or directly onto the foliage. In light of this, and with future vertical farming in mind, future inquiry should also include the application of H_2_ within hydroponic cultures.

It is extremely unlikely that plant cells perceive the presence of molecular hydrogen in a truly classical manner, by utilizing a cell receptor protein. Effects of H_2_ application have been seen on the levels of some reactive signalling molecules, particularly hydroxyl radicals and peroxynitrite, with other ROS and RNS being relatively unaffected [14]. Effects have also been reported on haem-oxygenase activity [76], whilst it has been postulated that the physical properties of H_2_ may be important in mediating HO-1 activity [16]. Certainly, much more work needs to be carried out to ascertain how H_2_ has effects on the biochemical processes inside cells. Many enzymes and regulatory proteins are redox sensitive, and the manner in which H_2_ interacts with the intracellular redox environment will need to be explored. Therefore, future work may also need to focus on exploring how H_2_ alters gene expression and the complement of proteins in cells. This may, of course, be different in distinct plant tissues, so work would need to be carried out on roots and leaves, for example, as well as using specialist cells within those tissues, such as guard cells.

It is reasonably well established that H_2_ has profound effects on plants, and can promote plant growth and development, and help to alleviate stress responses. Unlike ROS, NO, and H_2_S, which are all extremely toxic, (despite being used as signalling molecules) [13,85], molecular hydrogen, either as a gas or dissolved in water (HRW), is thought to be biologically safe [84]. Therefore, manipulation of the availability of molecular hydrogen to plants, and a full appreciation of the effects it elicits, along with an understanding of the underpinning mechanisms of action of H_2_, should be a priority in future plant science endeavors for such work is likely to enhance plant growth and crop yields in the future.

## Figures and Tables

**Table 1:** *S*-nitrosation sites in four hydrogenase proteins predicted using the iSNO-PseAAC prediction tool (http://app.aporc.org/iSNO-PseAAC/) [35]. Arabidopsis: ferredoxin hydrogenase (*Arabidopsis thaliana*) NP_567496.4; Medicago: protein NAR1 (*Medicago truncatula)* XP_003606579.2; Artemisia: ferredoxin hydrogenase (*Artemisia annua*) PWA71961.1; Human: cytosolic iron-sulfur assembly component 3 isoform 1 (*Homo sapiens*) NP_071938.1. Alignment table signifies that the regions are aligned in the amino acid sequences (highlighted in Figure 1). Target cysteine residues which may become -SNO are highlighted here in red. -SNO: nitrosated thiol.

Arabidopsis		Medicago		Artemisia		Human	
Position of -SNO	Sequence	Position of -SNO	Sequence	Position of -SNO	Sequence	Position of -SNO	Sequence
24	LNDFIAPSQACVISLKDSKPI	24	VNDFIVPSQACTVSLKERRLK	24	LNDYIAPSQGCVVSMKSGSDR		
64	VKISLKDCLACSGCITSAETV			68	VKISLKDCLACSGCITSAETV		
						179	FVRRFRGQADCRQALPLLASA
177	QSPLPVLSSACPGWICYAEKQ	176	KSSLPMISSACPGLICYAEKS	181	TSSLPMISSACPGWICYAEKT	190	RQALPLLASACPGWICYAEKT
182	VLSSACPGWICYAEKQLGSYV			186	MISSACPGWICYAEKTLGSYV	195	LLASACPGWICYAEKTHGSFI
233	HEVYHVTVMPCYDKKLEAARD	232	EEVYHVTVMPCYDKKLEASRD	237	GDIYHVTVMPCYDKKLEASRD	246	DKIYHVTVMPCYDKKLEASRP
						270	NQEHQTRDVDCVLTTGEVFRL
362	EGKTVLKFALCYGFQNLQNIV	366	DGETVLKFALCYGFSNLQKNI	365	EGKTVLKFALCYGFRNLQNVV		
380	NIVRRVKTRKCDYQYVEIMAC					385	NLVQRLKRGRCPYHYVEVMAC
394	YVEIMACPAGCLNGGGQIKPK						
						453	THWLQGTDSECAGRLLHTQYH

**Table 2:** A summary of some of the effects of molecular hydrogen in plants.

Plant Species Studies	Effects of H_2_ Studied	Comments	Reference(s)
Rice	Fitness parameters	Effects of roots and shoot length seenEffects on reproduction reported	[68]
Tomato	Lateral root formation	Effects mediated by NO	[75]
Cucumber	Adventitious root development	Effects mediated by haem oxygenase	[16]
Kiwifruit	Postharvest storage	Ripening and senescence was delayed	[8]
Alfalfa	Aluminum effects of roots	Mediated by NO	[76]
Rice	Germination in presence of aluminum	Effects on hormones and miRNA levels	[77]
Rice	Effects on hormone signalling		[78]
*Arabidopsis*	Salt tolerance	Mediated by altered antioxidants and sodium exclusion	[79]
Rice	Germination during salt stress	Alleviated oxidative stress. Antioxidants increased	[80]
*Medicago sativa*	Cadmium stress	Alleviated oxidative stress. Antioxidants increased	[6]
Alfalfa	Mercury stress	Alleviated oxidative stressRebalanced redox	[81]
Alfalfa	Paraquat induced oxidative stress	Alleviated oxidative stressMediated by haem oxygenase	[83]
